# The H_2_O_2_-dependent activity of a fungal lytic polysaccharide monooxygenase investigated with a turbidimetric assay

**DOI:** 10.1186/s13068-020-01673-4

**Published:** 2020-03-05

**Authors:** Frantisek Filandr, Petr Man, Petr Halada, Hucheng Chang, Roland Ludwig, Daniel Kracher

**Affiliations:** 1 BioCeV-Institute of Microbiology, The Czech Academy of Sciences, Prumyslova 595, 252 50 Vestec, Czech Republic; 2 Faculty of Science, Charles University, Hlavova 2030/8, 128 43 Prague 2, Czech Republic; 3 Biocatalysis and Biosensing Research Group, Department of Food Science and Technology, BOKU-University of Natural Resources and Life Sciences, Muthgasse 18, 1190 Vienna, Austria; 4 The University of Manchester, Manchester Institute of Biotechnology, Manchester, M1 7DN UK

**Keywords:** Lytic polysaccharide monooxygenase, Cellobiose dehydrogenase, Glucose oxidase, Hydrogen peroxide, Cellulose, *Neurospora crassa*

## Abstract

**Background:**

Lytic polysaccharide monooxygenases (LPMOs) are copper-dependent redox enzymes that cleave recalcitrant biopolymers such as cellulose, chitin, starch and hemicelluloses. Although LPMOs receive ample interest in industry and academia, their reaction mechanism is not yet fully understood. Recent studies showed that H_2_O_2_ is a more efficient cosubstrate for the enzyme than O_2_, which could greatly affect the utilization of LPMOs in industrial settings.

**Results:**

We probe the reactivity of LPMO9C from the cellulose-degrading fungus *Neurospora crassa* with a turbidimetric assay using phosphoric acid-swollen cellulose (PASC) as substrate and H_2_O_2_ as a cosubstrate. The measurements were also followed by continuous electrochemical H_2_O_2_ detection and LPMO reaction products were analysed by mass spectrometry. Different systems for the in situ generation of H_2_O_2_ and for the reduction of LPMO’s active-site copper were employed, including glucose oxidase, cellobiose dehydrogenase, and the routinely used reductant ascorbate.

**Conclusions:**

We found for all systems that the supply of H_2_O_2_ limited LPMO’s cellulose depolymerization activity, which supports the function of H_2_O_2_ as the relevant cosubstrate. The turbidimetric assay allowed rapid determination of LPMO activity on a cellulosic substrate without the need for time-consuming and instrumentally elaborate analysis methods.

## Background

LPMOs (CAZy AA9–11, 13–16) are copper-dependent redox enzymes that employ a redox reaction to cleave and decrystallize recalcitrant biopolymers [1, 2]. LPMO activity has been demonstrated in biomass-degrading bacteria [3], fungi [4] and, as of recently, also in firebrat (*Thermobia domestica*) [5], insect poxvirus [6] and the fern *Tectaria macrodonta* [7]. The substrate scope of LPMOs includes cellulose [8], in some cases soluble cello-oligosaccharides [9], chitin [3], starch [10] and various hemicelluloses [9, 11–13].

Since their discovery in 2010 [3], LPMOs have received ample attention in basic and applied research due to their synergistic interaction with hydrolytic enzymes [14, 15]. However, the insoluble nature of their substrates complicates the use of routine biochemical analysis methods, which typically require homogenous conditions. Furthermore, LPMOs depend on a steady supply of electrons and a cosubstrate while generating a complex array of oxidation products that necessitate specialized equipment for analysis. As a result, key questions on the LPMO catalytic cycle and kinetics, including the cosubstrate preference, await experimental clarification [16].

Despite their widespread distribution and their diverse substrate specificities, all known LPMOs share a highly conserved active site which includes a dyad of histidines coordinating a single Cu(II) atom [4, 17]. LPMO requires an external electron donor and an oxygen-containing cosubstrate for catalysis [16]. In fungi, electron-donating systems for LPMOs include a variety of phenols released during lignocellulose degradation [18, 19]. The fungal flavocytochrome cellobiose dehydrogenase (CDH) directly reduces the copper centre of LPMOs [20, 21]. Synergies with other fungal redox enzymes such as polyphenol oxidases [22], laccases [23] or oxidoreductases of the GMC-oxidoreductase family [24] were previously shown to provide a range of potential electron-donating systems for LPMOs through the release or recycling of phenolic lignin breakdown products. Potential electron-donating systems in other organisms, e.g. in bacteria or insects, await identification.

Reduced LPMOs are reported to utilize either O_2_ [3, 25] or H_2_O_2_ [26] as a cosubstrate, resulting in a monooxygenase or peroxygenase reaction, respectively. The outcome of both reactions is the regioselective insertion of an oxygen atom at the C1 [4] or C4 [9] carbon of the glycosidic linkage, which destabilizes and breaks the bond [17, 27]. Recent kinetic studies of bacterial [26, 28] and fungal LPMOs [29] showed that turnover numbers with H_2_O_2_ as cosubstrate exceeded those obtained with O_2_ by two orders of magnitude. A drawback of the peroxygenase reaction is the susceptibility of LPMOs for oxidative damage in the absence of substrate, or at high H_2_O_2_ concentrations [26, 30]. It was argued that the lower turnover with O_2_ could protect LPMO from such oxidation reactions and thus extend the operational stability to longer time-scales [29]. Despite several studies [4, 25, 26, 28, 29], it is still disputed whether O_2_ or H_2_O_2_ is preferred as cosubstrate in a natural environment. Here, it is worth noting that a number of GMC-oxidoreductases secreted by fungi also provide a steady H_2_O_2_ supply required for peroxidases involved in biomass degradation [31]. This includes CDH, which was shown to possess a weak oxidase activity [32] that can provide sufficient amounts of H_2_O_2_ for LPMO catalysis [33].

Typically, activity measurements for LPMOs rely on the identification of soluble, oxidized oligosaccharides, which are liberated by the LPMO [34]. Such studies are complicated by the array of possible oxidation products and the lack of suitable standards (e.g. C4-oxidized oligosaccharides). If C4-oxidizing LPMOs are used in combination with CDH, also doubly oxidized products occur, since CDH efficiently oxidizes the reducing end of soluble oligosaccharides [9]. Such analyses also miss the introduced carboxylic groups, resulting in aldonic acids in the insoluble fraction of the substrate, which make up a considerable fraction of the total reaction products (see e.g. [33]). Kuusk et al. [28] previously reported a detailed kinetic analysis of the chitin-active, bacterial LPMO CBP21 using ^14^C-labelled chitin. This procedure allowed for the sensitive detection of reaction products independent of their oxidation. A recently introduced activity assay for LPMOs is based on the colourimetric detection of a pyrocatechol violet–Ni^2+^ complex, which enabled quantifying the number of aldonic acids on the substrate generated by LPMO [35]. A drawback of this procedure is the inability to detect the activity of C4-oxidizing LPMOs, which do not introduce aldonic acids into the substrate. In homogenous solution, LPMO activity can be readily detected based on the quantification of H_2_O_2_ released in a futile side reaction that occurs in the absence of substrate [9, 36]. LPMOs also oxidize 2,6-dimethoxyphenol in the presence of peroxide and reducing equivalents, which results in the formation of the dimerization product coerulignone that can be quantified spectroscopically [37]. While these homogeneous assays may be used as a proxy for LPMO activity, they do not allow analysing reaction kinetics with native, heterogeneous LPMO substrates. To date, there is still the need for universal and easy-to-apply methods that enable measuring the time-dependent LPMO activity without specialized equipment.

Here, we employ a turbidimetric assay using a cellulose solution to examine the peroxygenase activity of the fungal, C4-oxidizing LPMO9C from the model fungus *Neurospora crassa.*

## Results

### LPMO activity monitored by a turbidimetric assay

Turbidimetry has been recently employed to screen the cellulolytic activity of a fungal LPMO towards phosphoric acid-swollen cellulose (PASC), which represents a disordered, amorphous form of cellulose. This screening assay measured the decrease in the optical density of the substrate after a defined incubation time of 360 min at 50 °C in microwell plates [38]. Here, we adapt this procedure into a continuous, turbidimetric assay to measure the time-dependent conversion of PASC by a cellulose-active LPMO.

**Fig. 1:**
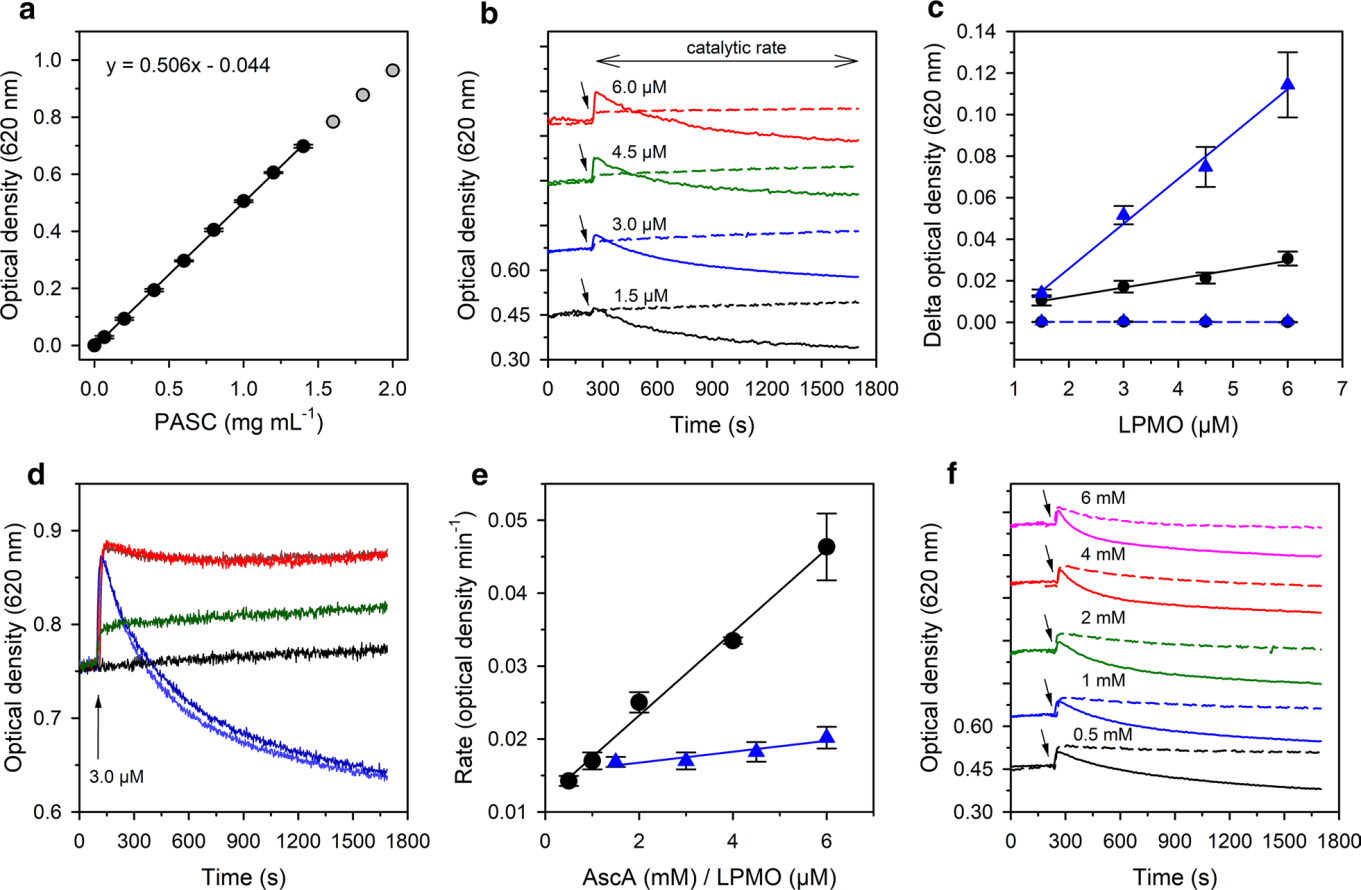
Activity of LPMO with ascorbate as reductant. **a** Calibration curve showing the relation between PASC concentration and the turbidity. Assays were performed in 50 mM sodium phosphate buffer, pH 6.0, at 30 °C. The equation for the linear range (0–1.4 mg mL^−1^ PASC) is given. **b** Time-dependent turbidity changes of a 0.8 mg mL^−1^ PASC solution incubated with various LPMO concentrations in the presence (solid lines) and absence (dashed lines) of 1 mM ascorbate. LPMO concentrations are specified above the traces. LPMO and ascorbate were added simultaneously to the assay (black arrows). Traces are vertically shifted by the same increment for better visibility. The extent of the catalytic reaction is indicated by an arrow above the graphs. **c** Absorbance changes observed in **b** upon titration of LPMO to PASC in the presence (blue triangles) or absence (black circles) of ascorbate. Dashed lines indicate the addition of LPMO to a buffer solution without PASC in the presence (blue triangles) or absence (black circles) of 1 mM ascorbate. Note that the curves coincide because no apparent changes in optical density were observed. **d** Degradation of PASC by LPMO measured in an anaerobic glove box. PASC (0.6 mg mL^−1^) was mixed with 3 µM *Nc*LPMO9C in the absence (green curve) or presence of 2 mM ascorbate (dark and light red curves). For comparison, the experiment was performed under the same conditions (3 µM LPMO and 2 mM ascorbate) under aerobic conditions (dark and light blue curves). A control containing only PASC (0.6 mg mL^−1^) is shown as a black curve. Note that for technical reasons a different photometer had to be used for this experiment. The equation for the linear range of the calibration curve (0–1 mg mL^−1^ PASC) was 1.16x + 0.03. The black arrow indicates the addition of LPMO and/or ascorbate. **e** Reaction rates determined from the initial slopes of the reactions shown in **b** (blue triangles) and **f** (black circles). **f** Reaction of LPMO (3 µM) with various concentrations of ascorbate (solid lines). The arrows indicate the addition of LPMO and ascorbate concentrations are specified above the traces. Identical reactions carried out in the presence of catalase (2000 U mL^−1^) are indicated as colour-coded dashed lines

Initially, we established the relation between PASC concentration and the loss of transmitted light intensity. The optical attenuation was linear up to a concentration of 1.4 mg mL^−1^ PASC (Fig. 1a). These measurements were performed under constant stirring to prevent the settling of particles in the suspension. In the standard assay, we employed a concentration of PASC (0.8 mg mL^−1^) that provided a stable baseline and a low background signal from the light scattering of larger substrate particles in the suspension. The molar concentration of PASC was 24.7 µM assuming an average chain length of 200 glucose units [39]. However, the particle distribution of PASC is not homogenous, which affects the depolymerization kinetics as discussed later. The reaction was started by injecting an LPMO-containing stock solution, which also contained the reducing agent. In experiments using H_2_O_2_ as the cosubstrate, the stock solution was added before addition of the H_2_O_2_. The optical density of the PASC suspension was continuously monitored at a wavelength of 620 nm, which was previously used for the turbidimetric measurement of cellulase activity [40].

### Binding of LPMO to PASC

In the following experiments, we employed LPMO9C from *Neurospora crassa* (*Nc*LPMO9C; UniProt accession number Q7SHI8), which is active on cellulose, hemicelluloses and soluble oligosaccharides [9, 11, 41]. This LPMO contains a family 1 carbohydrate-binding module (CBM1) which is fused to the catalytic domain via a lengthy linker peptide of 82 amino acids. In the first set of experiments, we employed 1 mM ascorbate, which is a commonly used concentration in LPMO conversion assays. The assay was started after 240 s by the addition of a relatively high concentration of LPMO (3 µM) to achieve a fast assay. Unexpectedly, this led to an instant increase in optical density within the mixing time (Fig. 1b). For both the reduced and the oxidized *Nc*LPMO9C, the optical density increased linearly with the enzyme concentration, but the observed increase was approximately three times higher for the reduced LPMO (Fig. 1c). The same increase in optical density was also observed when mixing ascorbate and LPMO under anaerobic conditions, demonstrating that this phase represents a non-catalytic reaction (Fig. 1d). Control experiments in the absence of PASC did not show detectable absorbance changes for all employed LPMO concentrations.

The fact that the reduced LPMO showed a higher increase in optical density than its oxidized form under both aerobic and anaerobic conditions suggests that the rapid initial increase in optical density is due to improved substrate binding. Previous binding experiments demonstrated a higher substrate affinity of *Nc*LPMO9C to PASC when the active site was in the reduced state [42]. In this study, the presence of ascorbate increased both the binding affinity and the binding capacity to PASC approximately twice [42]. A similar observation was made for the binding of LPMO9E from *Myceliophthora thermophila* to soluble oligosaccharides [43]. The binding of different substrate chains by the catalytic domain and the CBM1 under reducing conditions may lead to a “cross-linking” of PASC fibres and may thereby increase the optical density.

### Ascorbate-driven LPMO activity

Following the initial, very rapid increase in optical density, a second phase showing an attenuation of the signal was observed in assays containing LPMO and ascorbate (Fig. 1b). The decrease in optical density indicates the degradation of the PASC by the LPMO. To confirm catalysis, we mixed *Nc*LPMO9C with ascorbate in an anaerobic glove box (Fig. 1d) in the absence of any oxygen species. We observed the first phase of the reaction (binding of the LPMO to PASC), but found that the second, catalytic phase was completely suppressed. In the following, LPMO activity is expressed as the relative change in optical density per min. The rates were calculated from the linear slopes of the catalytic phase to avoid substrate depletion at the end of the experiment. An important and unexpected observation from these experiments is that almost similar reaction rates were obtained for different LPMO concentrations (Fig. 1e, blue triangles). The observed uncoupling of catalyst concentration and reaction rate—a fourfold increase of enzyme concentration correlated to a 25% increase of the activity—points towards a rate-limiting factor in the overall reaction. One reason could be the concentration of the reductant ascorbate, which was applied in a 1 mM concentration. We, therefore, varied the ascorbate concentration for 3 µM *Nc*LPMO9C (Fig. 1f). Initial rates calculated from these batch conversions demonstrated a strong correlation between activity and ascorbate concentration (Fig. 1e, black circles). A previous study that employed the bacterial *Sm*LPMO10A and chitin as the substrate showed a clear dependency of the LPMO reaction rate on the reductant concentration, with an apparent *K*_M_ for ascorbate of 2 µM [44]. However, it is also well documented that ascorbate can reduce O_2_ to H_2_O_2_ under commonly employed reaction conditions [24, 28]. Thus, providing a higher ascorbate concentration in the assays is likely to release higher amounts of H_2_O_2_, which can act as a cosubstrate for LPMO. To test whether the availability of H_2_O_2_ was the rate-limiting factor in the measurements, we replicated the activity assays in the presence of catalase (final concentration: 2000 U mL^−1^ at pH 6) to scavenge most of the formed H_2_O_2_. Under these conditions, we still observed the initial increase in optical density upon addition of LPMO, indicating that substrate binding of the LPMO was not compromised by the catalase. However, the subsequent catalytic reaction was clearly, but not fully suppressed in the presence of catalase (Fig. 1f, dashed lines).

### Interaction of *Nc*LPMO9C with *Nc*CDHIIA

**Fig. 2:**
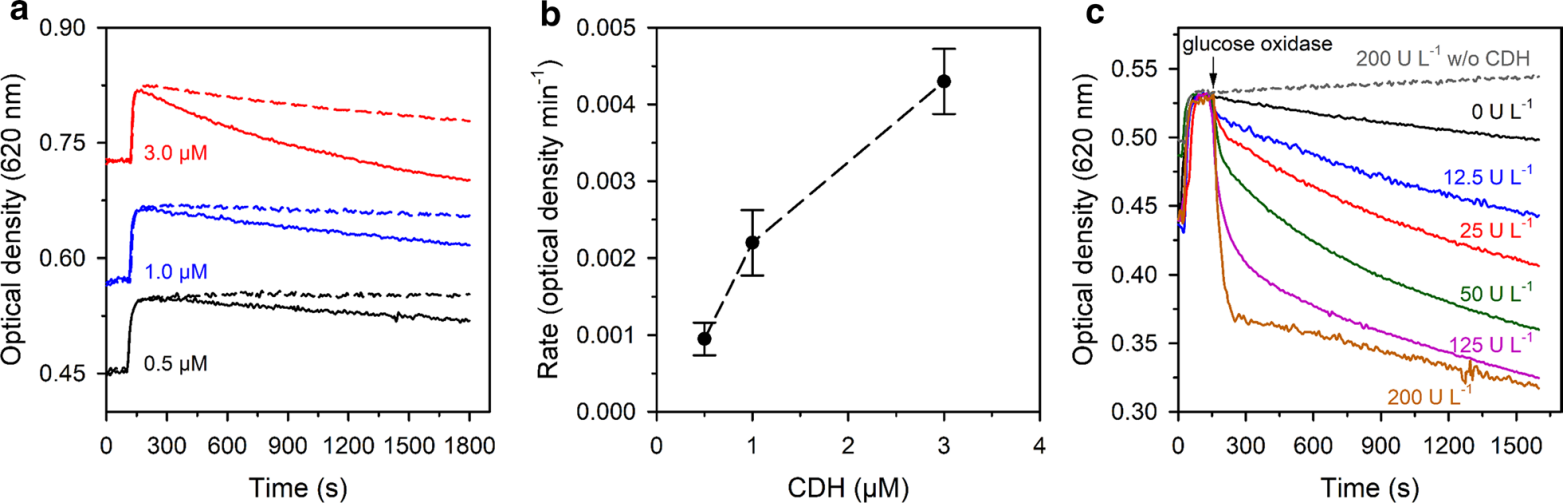
LPMO activity in the presence of CDH and glucose oxidase. **a** Solid lines indicate the time-dependent absorbance changes of a 0.8 mg mL^−1^ PASC solution in presence of 3 µM LPMO and 0.5, 1.0 or 3.0 µM *Nc*CDHIIA. Reactions were carried out in 50 mM potassium phosphate buffer, pH 6.0. The same reactions were carried out in presence of 2000 U mL^−1^ catalase (colour-coded dashed lines). Traces were vertically shifted for better visibility. **b** Reaction rates of PASC degradation of the reactions shown in **a**. **c** LPMO activity in the presence of 0.5 µM *Nc*CDHIIA and various indicated activities of glucose oxidase (GOX). The volumetric activity of GOX was determined as described in the “Methods” section. All samples contained 10 mM glucose and 10 mM lactose as a substrate for GOX and CDH, respectively, and were carried out at 30 °C in 50 mM sodium phosphate buffer, pH 6.0. In control experiments, *Nc*CDHIIA (grey dashed line) or GOX (black line) were avoided

We also initiated LPMO activity with cellobiose dehydrogenase (CDH), which is a proposed native interaction partner of LPMOs in wood-decaying fungi [20, 24]. CDHs oxidize cellobiose or soluble cello-oligosaccharides in an FAD-dependent reaction and reduce the LPMO active site via a dedicated, flexible cytochrome domain [21]. Reduced CDHs also have a low, FAD-dependent oxidase activity [45, 46], which can support LPMO activity through the slow release of H_2_O_2_ [33]. We used *Nc*CDHIIA (UniProt accession number Q7RXM0), the main secreted CDH in *N. crassa* [47], to activate *Nc*LPMO9C in the PASC turbidity assays (Fig. 2a). The activity of LPMO in this reaction setup was strictly dependent on the presence of cellobiose as CDH substrate (Additional file 1: Figure S1). *Nc*CDHIIA in combination with cellobiose induced moderate LPMO activity, which was dependent on the applied *Nc*CDHIIA concentration. The observed rates were approximately one order of magnitude lower than those obtained with ascorbate as LPMO-reductant (Figs. 2b vs 1e). Catalase (2000 U mL^−1^) completely inhibited the reaction at a low, 0.5 µM concentration of *Nc*CDHIIA, while at higher concentrations a weak LPMO activity was observed, possibly reflecting the incomplete H_2_O_2_ removal by the catalase. The obvious inhibition of the turbidimetric PASC assay by catalase at low CDH concentrations indicates that H_2_O_2_ was predominantly used as cosubstrate by *Nc*LPMO9C. Since both *Nc*LPMO9C and *Nc*CDHIIA feature a CBM1 that binds to cellulose, the spatial proximity of the two enzymes during catalysis, which is required for the electron transfer between both enzymes, may also provide a locally increased H_2_O_2_ concentration in the vicinity of the heterogeneous substrate.

To further probe the effect of H_2_O_2_ on CDH-driven LPMO activity, we used commercial glucose oxidase (GOX) from *Aspergillus niger* for the in situ generation of H_2_O_2_. GOX in combination with glucose and LPMO did not lead to changes in the optical density (Fig. 2c), demonstrating that an LPMO-specific reductant is required to induce activity. For LPMO reduction a low, 0.5 µM concentration of *Nc*CDHIIA in combination with 10 mM cellobiose was added. Under these conditions, the addition of GOX led to a rate enhancement that depended on the applied GOX activity and, therefore, also on the amount of produced H_2_O_2_. At high GOX activities, a fast, initial attenuation of the optical density was followed by a slower phase of signal decay. This indicates a rapid deactivation of *Nc*LPMO9C at high GOX concentrations, possibly due to H_2_O_2_-induced oxidation of the copper-coordinating amino acids [26]. Such deactivation effects were recently observed for a bacterial LPMO, which was rapidly deactivated when the H_2_O_2_ supply exceeded the enzyme’s capability to convert the cosubstrate [33]. The pronounced rate acceleration upon addition of GOX in the presence of a low, 0.5 µM concentration of *Nc*CDHIIA indicated that not the availability of reducing equivalents, but the H_2_O_2_ concentration was the rate-limiting factor in these reactions.

**Fig. 3:**
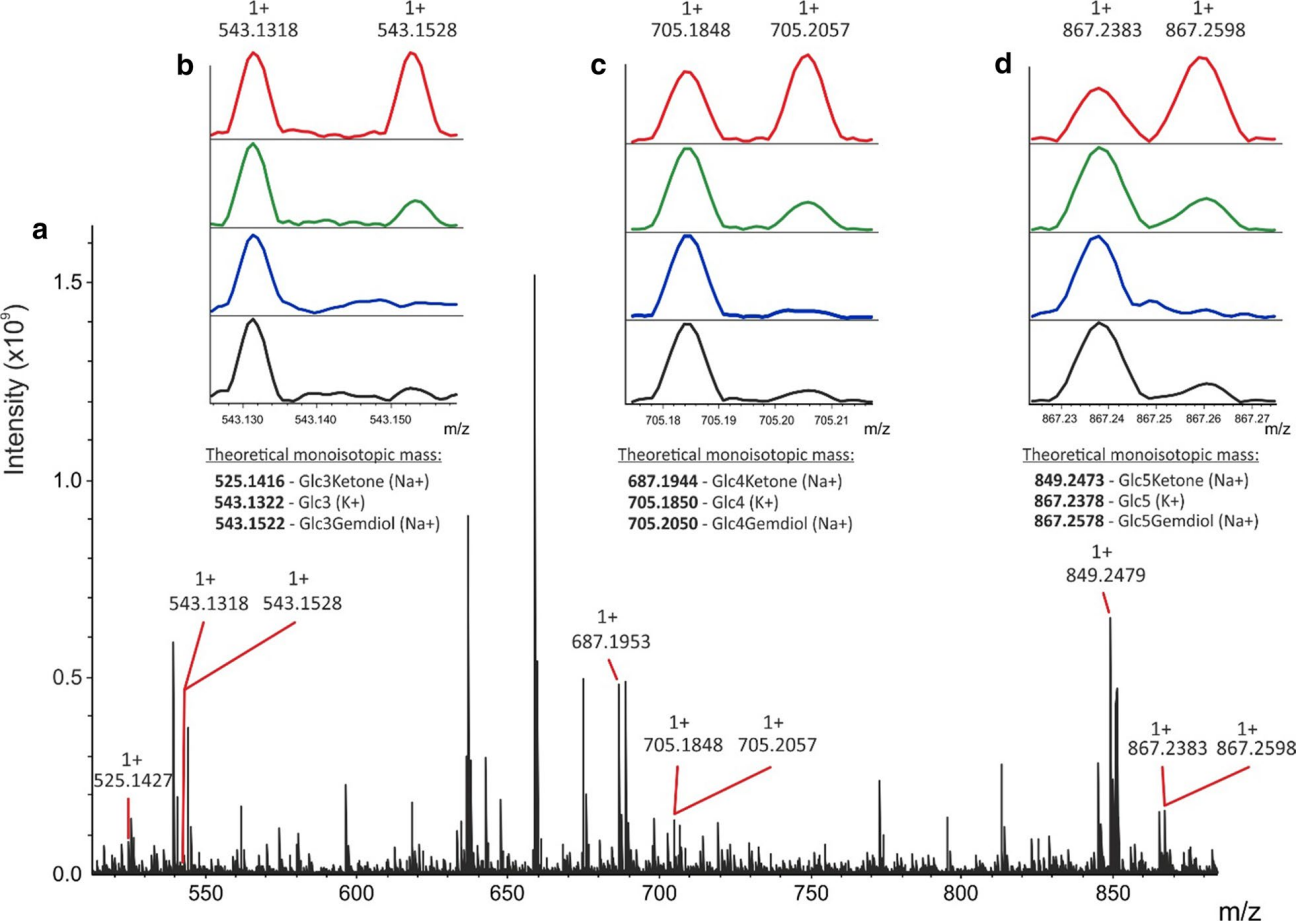
MALDI-MS analysis of oxidized oligosaccharide products. Reactions containing 10 mg mL^−1^ PASC, 3 µM LPMO, 0.1 µM CDH, 1 mM lactose and H_2_O_2_ (120 µM final concentration added in four batches of 30 µM over 30 min) were analysed under different conditions using MALDI-MS. **a** Overview of selected parts of the MALDI-MS spectra obtained from samples containing all components (PASC, CDH, lactose, LPMO and H_2_O_2_) showing various oligosaccharide products. **b**–**d** Details of selected product spectra under four conditions: PASC with oxidized LPMO (black line), PASC with oxidized LPMO and H_2_O_2_ (blue line), PASC with CDH, lactose and LPMO (green line) and PASC with CDH, lactose, LPMO and H_2_O_2_ (red line). Addition of H_2_O_2_ led to a notable, relative increase in C4-oxidized products compared to the unoxidized forms of the analysed oligosaccharides. All oligosaccharides and their oxidized products were detected as sodium or potassium adducts. Shown are also the theoretical monoisotopic masses of all analysed reaction products. Peaks with no labels are matrix adducts or other, non-carbohydrate based, background signals

To verify that the observed increase in activity upon H_2_O_2_ addition detected by turbidimetry corresponds to the formation of oxidized oligosaccharide products, MALDI-MS measurements were performed on the soluble fraction of the reaction mixtures. The formation of products was followed in reactions containing PASC, LPMO, CDH and lactose and in related reactions spiked several times with H_2_O_2_ during the course of the incubation (Fig. 3). C4 oxidized products, which are typical products of the *Nc*LPMO9C reaction [9], were detected in the form of sodium adducts of C4 ketones and geminal diols. Small amounts of native (unoxidized) oligosaccharides, e.g. Glc3, Glc4 and Glc5, were also present in control samples containing only PASC, CDH and lactose. Such products may also occur during the LPMO action due to a weak hydrolytic background [48]. While absolute quantitation cannot be achieved by MALDI-MS, the changes in the ratio of unoxidized and oxidized oligosaccharides between the individual conditions clearly indicated the boosting effect of H_2_O_2_ on the action of *Nc*LPMO9C (Fig. 3b–d).

The high resolving power and high mass accuracy of the FT-ICR MS allowed us to unambiguously assign different carbohydrate molecules and their adduct state. For example, we were able to clearly distinguish between Glc(n)(K+) and Glc(n)Gemdiol(Na^+^) adducts, which differ only by 0.02 Da. The mass measurements can also provide indirect proof whether the LPMO generates C1 or C4 oxidized products. C1 oxidation leads to the formation of sugar lactones, which undergo conversion into aldonic acids. The acidic products are then preferentially detected in the form of salt (sodium or potassium), charged by an additional alkali metal cation (Na^+^ or K^+^) [27, 34]. On the other hand, C4 oxidizing LPMOs create keto/gemdiol forms, which are not forming salts and are present only as single alkali metal cation charged masses. Since we have not detected aldonic acids in any of the reaction mixtures and only detected gemdiols, we can conclude that the *Nc*LPMO9C indeed generated C4 oxidation products.

### The peroxygenase reactivity of LPMO

**Fig. 4:**
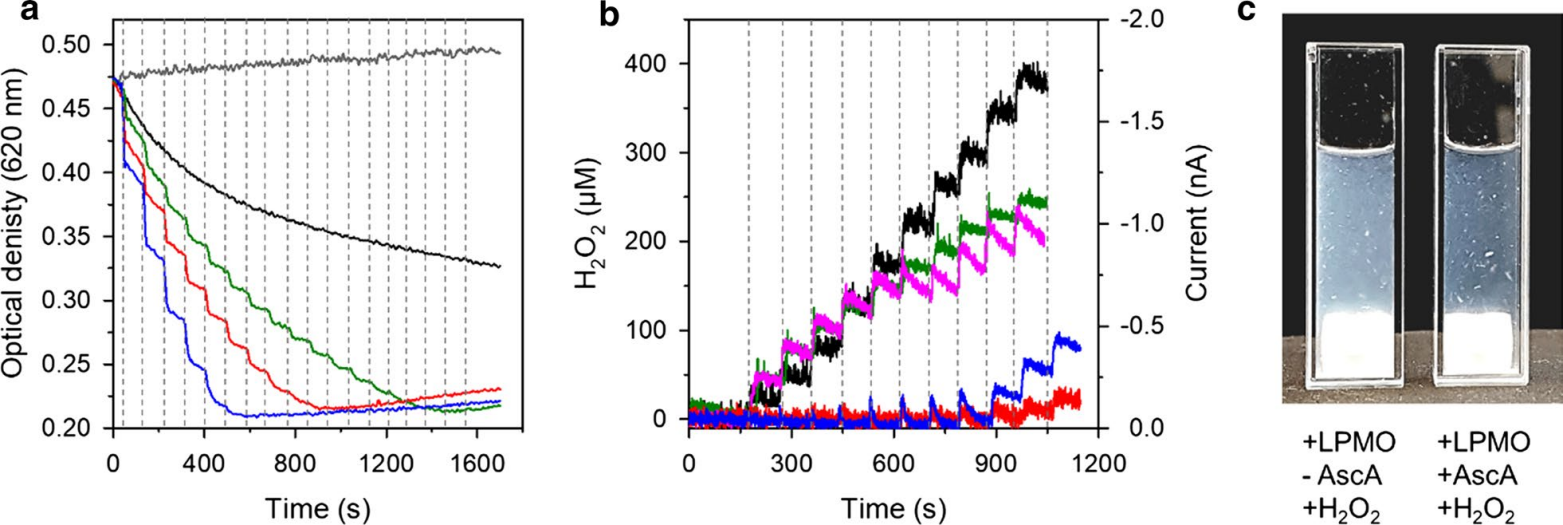
Effect of H_2_O_2_ on the degradation of PASC by LPMO. **a** Sequential titration of 20 µM (green line), 40 µM (red line) or 80 µM (blue line) H_2_O_2_ to a solution containing 0.8 mg mL^−1^ PASC, 3 µM LPMO and 2 mM ascorbate. The background reaction of LPMO in the presence (black line) or absence (grey line) of 2 mM ascorbate. **b** Time-dependent, electrochemical measurement of H_2_O_2_. All reactions were carried out at a volume of 12 mL under constant stirring at 30 °C and contained 0.8 mg mL^−1^ PASC in 50 mM sodium phosphate buffer, pH 6.0. Vertical dotted lines indicate the addition of fresh H_2_O_2_ which was added approximately every 90 s in aliquots of 40 µM. Shown is the addition of H_2_O_2_ to PASC (black line); PASC and 3 µM LPMO (green line); PASC and 2 mM ascorbate (magenta line); PASC, 2 mM ascorbate and 3 µM LPMO (red line). In an additional experiment, PASC, 2 mM ascorbate and 3 µM LPMO was supplemented with 80 µM H_2_O_2_ per addition (blue line). **c** Degradation of 0.8 mg mL^−1^ PASC by 3 µL LPMO. H_2_O_2_ (40 µM) was sequentially added to the samples every 90 s in presence or absence of 2 mM ascorbate. The total amount of added H_2_O_2_ was 400 µM

To determine the H_2_O_2_ consumption rate by LPMO, we tested the reactivity of *Nc*LPMO9C with H_2_O_2_ by titrating aliquots of H_2_O_2_ to reactions containing 0.8 mg mL^−1^ PASC, 3 µM LPMO and 2 mM ascorbate. In these experiments, H_2_O_2_ was added to the reaction every 90 s using three different concentrations (20, 40 or 80 µM per addition). The total change in the reaction volume due to the addition of H_2_O_2_ was less than 3% in all assays. The addition of H_2_O_2_ to reduced LPMO caused an immediate decrease in optical density, which points towards a fast consumption of H_2_O_2_. This reaction was much faster than the reference reaction without H_2_O_2_ (Fig. 4a). The substrate conversion rate could not be determined because it was as fast or faster than the mixing time of the cuvette (ca. 10 s). However, doubling the amount of added H_2_O_2_ also doubled the observed change in optical density. For all titrations, approximately 350–400 µM of H_2_O_2_ was required to reach maximal observable changes, corresponding to approximately 0.2 units of optical density. Addition of H_2_O_2_ or LPMO beyond this lower limit did not induce further changes in the optical density of the PASC suspension. Control experiments in which either LPMO or reductant were omitted did not show any changes in the optical density of the PASC suspension (Additional file 1: Figure S2). Likewise, the titration of H_2_O_2_ to oxidized *Nc*LPMO9C had no observable effect on the optical density of the PASC (Additional file 1: Figure S2).

To correlate the observed substrate degradation with the cosubstrate consumption, we followed the depletion of H_2_O_2_ using electrochemical detection of H_2_O_2_ (Fig. 4b). These assays were carried out at a larger volume of 12 mL in a stirred electrochemical cell to avoid exceeding consumption of H_2_O_2_ by the electrode. Titration of 40 µM H_2_O_2_ to reactions containing only PASC or only LPMO showed a stable, H_2_O_2_ concentration-dependent decrease of the measured current. The addition of H_2_O_2_ to oxidized LPMO resulted in slightly lower currents, indicating H_2_O_2_ depletion through a background reaction. Under these conditions, no turbidimetric changes of PASC were observed (Additional file 1: Figure S2) showing that this futile reaction did not induce observable catalytic events. The addition of H_2_O_2_ to reactions containing 2 mM ascorbate (Fig. 4b, magenta line) led to a slow depletion of H_2_O_2_, possibly via reduction of the H_2_O_2_ [49, 50]. Upon titration of H_2_O_2_ to a reaction containing ascorbate, LPMO and PASC, no detectable increase in the H_2_O_2_ concentration was observed, showing that H_2_O_2_ was rapidly consumed in this experiment (Fig. 4b, red line). This is a clear indication that the consumption of the cosubstrate by the system occurred within the response time of the electrochemical sensor, which was approximately 3 s. After 9 H_2_O_2_ additions, corresponding to a total added H_2_O_2_ concentration of 360 µM, a built-up of H_2_O_2_ was observed. This concentration coincides with the required H_2_O_2_ concentration that induced maximal changes in optical density of PASC in degradation assays carried out under comparable conditions (Fig. 4a, red line). Doubling the concentration of added H_2_O_2_ to 80 µM per addition (Fig. 4b, blue line) resulted in notable signal spikes after 4–5 additions (320–400 µM), which compares well to the experiments shown in Fig. 4a which employed the same H_2_O_2_ addition rate. Taken together, these experiments demonstrate fast consumption of H_2_O_2_ by an LPMO-dependent reaction and connect the observed absorbance changes to the consumption of H_2_O_2_. The visual change that accompanied the degradation of PASC by LPMO upon titration with 40 µM H_2_O_2_ is shown in Fig. 4c. The images suggest that, to a large extent, *Nc*LPMO9C preferentially targeted finely dispersed, amorphous PASC while bigger particles remained largely intact at the end of the reaction. The heterogeneity of the substrate may also explain why the reaction levelled off at a certain optical density.

### Substrate binding of LPMO during H_2_O_2_-mediated PASC degradation

**Fig. 5:**
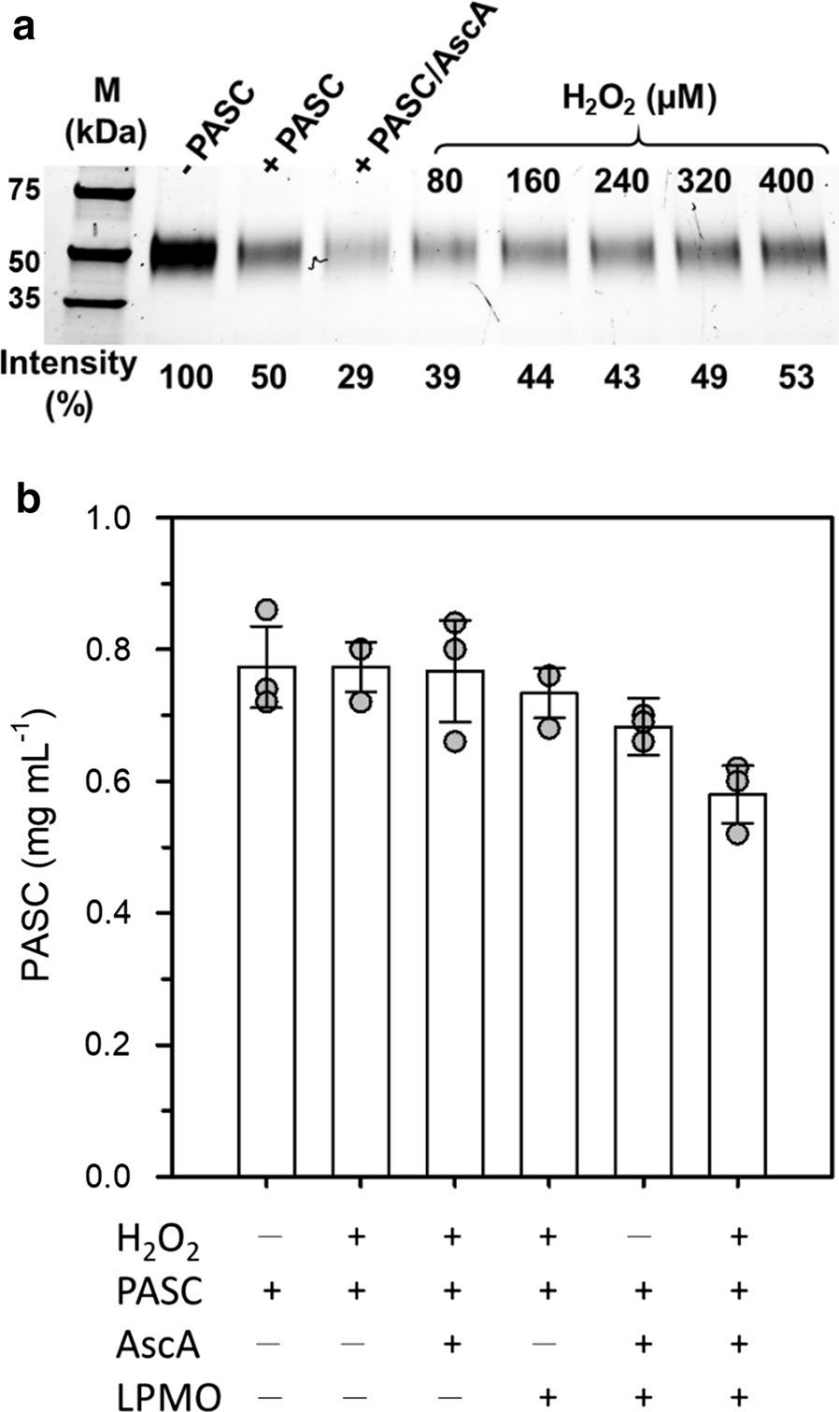
Binding of *Nc*LPMO9C to PASC and substrate degradation during H_2_O_2_-mediated PASC degradation. **a** SDS-PAGE analysis of supernatants obtained from titration of reduced *Nc*LPMO9C with aliquots of 40 µM H_2_O_2_ (Fig. 4a, red line). Three µM LPMO was incubated for 10 min in buffer solution (“-PASC”) or together with 0.8 mg mL^−1^ PASC (“+PASC”). To the latter reaction mix, ascorbate was added to a final concentration of 2 mM (“+PASC/AscA”), and titration with H_2_O_2_ in aliquots of 40 µM was performed. The intensity (in %) denotes the intensities of the bands relative to those of *Nc*LPMO9C incubated in buffer solution. Intensities were calculated with the Image Lab Software Suite (BioRad). All reactions were carried out in 50 mM potassium phosphate buffer, pH 6.0. The SDS-PAGE analysis was repeated twice with the error being within 5%. **b** Weight determination of PASC in different control experiments. The total concentration of H_2_O_2_ added to the samples was 400 µM, which was added in aliquots of 40 µM every 90 s (as shown in Fig. 4a, red line). All assays were performed in 3 mL quartz cuvettes at 30 °C and under constant stirring. For each data point shown, 2 reactions containing 2.5 mL of sample solution were pooled, centrifuged and washed twice with deionized water. The dry weight of PASC in each reaction mix was determined after drying the sample at 55 °C to a constant weight (ca. 16 h of incubation)

To gain further insight into the binding of LPMO to PASC, we monitored the fraction of free *Nc*LPMO9C during the titration of reduced LPMO with 10 aliquots of 40 µM H_2_O_2_. Samples of 50 µL were regularly withdrawn from this reaction and the supernatants analysed by SDS-PAGE after centrifugation (Fig. 5a). Incubation of LPMO with PASC in absence of reductant reduced the concentration of soluble LPMO by 50%, indicating binding of the other 50% of LPMO to the substrate. Addition of ascorbate to this reaction instantly increased the fraction of bound enzyme to 71%. This compares well to the observed changes in optical density in Fig. 1b, which showed a higher signal change for the reduced LPMO when compared to the oxidized enzyme. The fraction of free enzyme gradually increased upon titration with H_2_O_2_ (Fig. 5a). Quantitative assessment of PASC by weight determination (Fig. 5b) showed that notable substrate degradation occurred only in samples containing ascorbate together with LPMO. Addition of H_2_O_2_ to this mixture led to a notably higher PASC degradation than observed in the presence of ascorbate alone. In this reaction, approximately 20% of the PASC initially present in the assay was solubilized by the LPMO. In the same reaction, the optical density of PASC decreased by ca. 45% (from 0.47 to 0.21 optical density at 620 nm). Thus, part of the observed absorbance changes may be a result of PASC modification rather than solubilization, e.g. via the introduction of oxidized ends, or the release of insoluble oligomers. Results obtained from bacterial or fungal LPMOs previously showed that only approximately 50% of the total introduced oxidized ends were found on soluble oligomers, while the remaining modifications occurred on the insoluble fraction [33].

## Discussion

A growing body of evidence demonstrates that LPMOs use H_2_O_2_ as cosubstrate with a much higher catalytic efficiency than O_2_ [26, 28, 44, 51]. While the cosubstrate preference of LPMOs in their native environments is still debated [29] the efficient peroxygenase reactivity may be beneficial in industrial settings to achieve faster biomass depolymerization [52].

The activity of LPMOs is typically assessed in the presence of an about 1 mM concentration of ascorbate, which reduces the active-site copper and initiates the oxidative degradation of the substrate. Several recent publications, however, raised the question whether the observed activity is due to an O_2_-dependent monooxygenase reaction, or, at least partially, depends on the H_2_O_2_ that is slowly released by the reaction of oxygen with the reductant ascorbate [26, 31, 44]. In addition, reduced LPMOs in solution may also release low H_2_O_2_ concentrations via an uncoupling reaction [36]. Results obtained with the turbidimetric assay support an H_2_O_2_-dependent LPMO activity. First, we observed that the rate of *Nc*LPMO9C increased linearly with the concentration of ascorbate. While we cannot exclude experimentally that the assays may not have been carried out under saturating ascorbate concentrations, a recent study showed that the bacterial LPMO10A from *Serratia marcescens* had an apparent *K*_M_-value of 2 µM for ascorbate [44]. Even if the *K*_M_-value of *Nc*LPMO9C for ascorbate would be 50-times higher, the high 0.5–6 mM ascorbate concentration present in our assays should still provide sufficiently saturating conditions to achieve maximal turnover. The reduction of the active site by ascorbate is not the rate-limiting step in the overall LPMO reaction at high ascorbate concentrations [53] and providing more reducing equivalents should not exert a boosting effect on the LPMO catalysis. From experiments with the H_2_O_2_ scavenger catalase, we conclude that the H_2_O_2_ generated from the oxidation reaction of O_2_ by ascorbate is preferentially used as cosubstrate by the *Nc*LPMO9C for the degradation of the cellulose substrate. Stability measurements of ascorbate conducted under the same reaction conditions used in this study (50 mM phosphate buffer, pH 6, and 30 °C) showed that a concentration of 1 mM ascorbate depleted within 100 min of incubation (Figure S10 in Ref. [24]), forming H_2_O_2_ and dehydroascorbic acid as the degradation products.

We also found that the reaction of LPMO with the native electron donor cellobiose dehydrogenase depended on the presence of H_2_O_2_. The CDH/LPMO system was sensitive to the presence of catalase, which is in good agreement with a previous report showing that a CDH variant with enhanced oxygen reactivity was more efficient in initiating the activity of a bacterial LPMO [33]. In this study, the measured LPMO reaction rates corresponded to the rate of H_2_O_2_ formation by CDH, while the electron transfer from CDH to LPMO was not rate-limiting. Here, we confirm and extend this observation by demonstrating that the same effects occur when using a CDH together with an LPMO from the same organism (*N. crassa*) during the degradation of a cellulosic substrate. Experiments using a low amount (0.5 µM) of CDH showed that the LPMO reaction rate could be tuned by the addition of glucose oxidase/glucose, indicating that reductive activation of the LPMO by CDH was not rate-limiting.

Also, it should be noted that the high CDH concentrations (0.5–3 µM) employed in our assays aimed at visualizing degradation effects within the assay time of ~ 30 min, but may not reflect conditions encountered in vivo. Quantitative secretome analysis of the fungus *N. crassa* previously showed that *Nc*CDHIIA constituted only a minor fraction of the proteins detected under cellulolytic conditions (2.4% or 0.28 µmol g^−1^ secretome) [54]. In comparison, the 3 LPMOs identified in this study together made up 14.6% of the total secretome, corresponding to 5.23 µmol g^−1^ secretome. This indicates that a 15- to 20-fold lower concentration of CDH is used by the fungus to support LPMO activity.

Overall, the herein used assay procedure allows a rapid determination of LPMO activity under heterogeneous conditions. We reason that the limits of our assay were largely determined by substrate depletion due to the modification or depolymerization of PASC. Conversion experiments carried out at different H_2_O_2_ feeding rates all converged at a similar optical density (Fig. 4a). However, the addition of fresh PASC, ascorbate or H_2_O_2_ at the end of the assays (after addition of 400 µM H_2_O_2_) did not induce notable absorbance changes of the PASC solution (Additional file 1: Figure S3). We, therefore, conclude that a limitation of binding sites on PASC and oxidative damage of the unbound LPMO [26] are the limiting factors of this assay procedure.

## Conclusions

Lytic polysaccharide monooxygenases employ a unique redox mechanism to degrade recalcitrant polysaccharides. To date, there is still an ongoing dispute whether O_2_ or H_2_O_2_ is the preferred cosubstrate of the enzyme. Using different reducing systems, including the native reductase cellobiose dehydrogenase, we here show that the depolymerization of a cellulosic substrate by *Nc*LPMO9C depends on the supply of H_2_O_2_. Furthermore, we introduce an easy-to-apply assay for lytic polysaccharide monooxygenases that employs an insoluble cellulose substrate.

## Methods

### Enzymes and chemicals

Cellobiose dehydrogenase IIA (CDHIIA) and lytic polysaccharide monooxygenase 9C (LPMO9C) from *N. crassa* were recombinantly produced in *Pichia pastoris* X-33 cells as previously reported [55]. Purification was done by sequential hydrophobic interaction chromatography (HIC) and anion exchange chromatography (AIEX) [36, 55]. The purity of the enzymes was verified by SDS-PAGE and activity assays.

CDH activity was measured spectrophotometrically by monitoring the reduction of the FAD-dependent electron acceptor dichlorophenol indophenol (DCIP, *ε*_520_ = 6.8 mM^−1^ cm^−1^) or the heme *b*-dependent chromogen cytochrome *c* (cyt *c*, *ε*_550_ = 19.6 mM^−1^ cm^−1^). Assays had a total volume of 1 mL and contained 30 mM lactose as CDH substrate along with 300 µM DCIP or 20 µM cyt *c* in 50 mM potassium phosphate buffer, pH 6.0. One unit of CDH activity was defined as the amount of enzyme that reduced 1 µmol of the electron acceptor per min under the given assay conditions.

Catalase from *Corynebacterium glutamicum* and FAD-dependent glucose oxidase from *A. niger* were obtained from Sigma Aldrich and used without additional purification. Catalase activity was assayed by monitoring the decrease of 40 mM H_2_O_2_ at a wavelength of 240 nm (*ε*_240_ = 43.6 M^−1^ cm^−1^ [56]). Assays had a total volume of 1 mL and were performed at 30 °C in 50 mM potassium phosphate buffer, pH 6.0. One unit of catalase activity was defined as the amount of enzyme consuming 1 μmol H_2_O_2_ per min.

Glucose oxidase activity was assayed with a peroxidase-coupled assay using ABTS [2,2′-azinobis(3-ethylbenzthiazolinesulfonic acid)] (*ɛ*_420_ = 36 mM^−1^ cm^−1^) as the chromogenic substrate. Assays had a total volume of 1 mL and contained 10 mM glucose, 10 mM ABTS and 7 U mL^−1^ horseradish peroxidase II (Sigma Aldrich) in 50 mM potassium phosphate buffer, pH 6.0. One unit of glucose oxidase activity was defined as the amount of enzyme necessary for the generation of 1 μmol of H_2_O_2_ per min.

### Preparation of phosphoric acid-swollen cellulose (PASC)

Phosphoric acid-swollen cellulose (PASC) was prepared by dissolving 8 g of microcrystalline cellulose (20–160 µm) in 200 mL of ice-cold 85% (v/v) phosphoric acid. The solution was stirred for 1 h at 4 °C. After removing undissolved cellulose, 1.8 L of ice-cold HQ-water was added to induce the precipitation of PASC. The precipitate was washed on a vacuum pump with deionized water (ca. 2.0 L), with 2 L of a 2 M sodium bicarbonate solution and finally with 50 mM potassium phosphate buffer, pH 6.0, until a constant pH was measured. Before utilization, PASC was homogenized with a disperser (Ultra Turrax, Ika).

### Turbidimetric measurement of PASC and determination of LPMO activity

LPMO activity was measured based on the decrease of the optical density of a PASC suspension upon degradation [40]. The optical density of PASC was determined at 620 nm using a temperature-controlled, single-beam UV–visible spectrophotometer (U-3000, Hitachi) with a built-in magnetic stirrer. The measurement setup consisted of a quartz cuvette with 3 mL volume containing a 6 mm cross-shaped magnetic stirrer. The cuvette was filled with 2.5 mL of the PASC suspension and was placed in a temperature-controlled UV–Vis spectrometer (Hitachi U-3000). The stirrer speed was set to an angular frequency of approximately 50 rad s^−1^ and the PASC suspension was equilibrated within the instrument for 10 min at 30 °C. The time to achieve uniform mixing in the cuvette was approximately 10 s. The linear relation between the PASC concentration and its optical density at 620 nm was verified between 0 and 1.4 mg PASC mL^−1^ (Fig. 1a). Standard activity assays contained 0.8 mg mL^−1^ PASC and 3 µM of LPMO. Reducing agents for LPMO were ascorbate, or *Nc*CDHIIA together with 10 mM cellobiose. All assays were performed at 30 °C unless stated otherwise. Control experiments were performed by adding only ascorbate or *Nc*LPMO9C to PASC. The activity was assessed based on the initial, linear decrease in optical density by fitting the data to a linear equation. PASC degradation experiments in absence of oxygen were performed in an anaerobic glove box (Whitley DG250, Don Whitley Scientific) which was continuously flushed with a nitrogen/hydrogen mixture (99:1). Residual oxygen traces were removed by a palladium catalyst and the generated water vapour captured by silica gel. Measurements were performed on an Agilent 8453 UV–visible spectrophotometer equipped with a magnetic stirrer. During all measurements, the temperature inside the glove box was maintained at 25 ± 1 °C by an external thermostat.

### Matrix-assisted laser desorption/ionization mass spectrometry (MALDI-MS) analysis

MALDI-MS analysis was performed on a Bruker SolariX 15T FT-ICR mass spectrometer. PASC was washed two times with 250 mM sodium acetate, centrifuged at 2000×*g* for three minutes, and resuspended in 25 mM TRIS, pH 6.0, at a concentration of 10 mg mL^−1^. *Nc*CDHIIA (0.1 µM), lactose (1 mM) and *Nc*LPMO9C (3 µM) were added to a total reaction volume of 500 µL. The reaction mixture was incubated for 30 min at 30 °C under constant shaking. H_2_O_2_ was added to a concentration of 30 µM (5 µL of a 3 mM H_2_O_2_ stock solution) at the start of the incubation and after 10, 20 and 30 min resulting in a total added concentration of 120 µM H_2_O_2_ at the end of the experiment. Samples were taken at the end of the incubation, desalted using a porous graphitic carbon resin (HyperCarb, Thermo Fisher Scientific) in a pipette tip (washed with water and eluted with 50% ACN) and were spotted (1.5 µL) on a MALDI plate in a 10, 20 and 60 µg µL^−1^ DHB matrix in 30% ACN (1.5 µL). Measured values are a sum of 1500 laser shots randomly distributed across the sample spot. Results are only shown for 20 µg µL^−1^ matrix that yielded the highest intensities of the products.

### Electrochemical measurements

Chronoamperometric measurements were performed in a water-jacketed electrochemical cell filled with 12 mL of sample solution connected to a water bath (Julabo F12, Germany) using an Autolab PGSTAT204 potentiostat (Metrohm, Netherlands). A standard three-electrode configuration employed a platinum disk microelectrode with a diameter of 100 μm as the working electrode, an Ag/AgCl electrode as the reference electrode and a platinum coiled wire as the auxiliary electrode (BAS Inc.). Prior to all measurements, the phosphate buffer solution (50 mM, pH 6.0) containing 0.8 mg mL^−1^ PASC, 2 mM ascorbate and 3 µM *Nc*LPMO9C was degassed by bubbling with nitrogen for 20 min and subsequently protected by applying a nitrogen atmosphere during the whole measurements. A potential of − 0.15 V was applied to detect H_2_O_2_. When the background current reached a stable signal, the freshly prepared and degassed H_2_O_2_ sample was injected into the PASC suspension through an FEP tube (diameter 0.15 mm) connected to a 1-mL syringe (SGE Analytical Science). All measurements were conducted at 30.0 ± 0.2 °C and a magnetic stirrer operated at an angular frequency of approximately 50 rad s^−1^ provided convective transport. The data were collected at 0.5 s^−1^ and corrected for the background current.

## Supplementary information

**Additional file 1: Figure S1.** Incubation of 3 µM LPMO and 0.8 mg mL^−1^ PASC with CDH at concentrations of 0.5 µM (red) 1 µM (blue) or 3 µM (green) in absence of cellobiose. Black line: 3 µM CDH and 10 mM cellobiose in absence of LPMO. All reactions were carried out under constant stirring at 30 °C in 50 mM sodium phosphate buffer, pH 6.0. **Figure S2.** Titration of oxidized LPMO (3 µM) and 0.8 mg mL^−1^ PASC with 20 µM (green), 40 µM (red) or 80 µM (blue) H_2_O_2_ (solid lines). Dashed, coloured lines show the titration of 2 mM ascorbate and 0.8 mg mL^−1^ PASC with 20 µM (green), 40 µM (red) or 80 µM (blue) H_2_O_2_. The vertical dashed lines indicate the addition of H_2_O_2_. The arrow indicates the addition of LPMO (solid lines) or 2 mM ascorbate (dashed lines). All reactions were carried out under constant stirring at 30 °C in 50 mM sodium phosphate buffer, pH 6.0. **Figure S3.** Titration of LPMO (3 µM) and 0.8 mg mL^−1^ PASC with 40 µM H_2_O_2_. The vertical dashed lines indicate the addition of H_2_O_2_. The arrow indicates the addition of fresh PASC which was either added alone (green line) or simultaneously with 1 mM ascorbate (AscA, black line). The blue line indicates the addition of ascorbate (1 mM). All reactions were carried out under constant stirring at 30 °C in 50 mM sodium phosphate buffer, pH 6.0.

## Data Availability

The datasets used and/or analysed during the current study are available from the corresponding author on reasonable request.

## Abbreviations

LPMO: Lytic polysaccharide monooxygenase
CDH: Cellobiose dehydrogenase
CBM: Carbohydrate-binding module
PASC: Phosphoric acid-swollen cellulose
ANS: 8-Anilinonaphthalene-1-sulfonic acid
EDTA: Ethylenediaminetetraacetic acid
DHB: 2,5-Dihydroxybenzoic acid
TCEP: Tris(2-carboxyethyl)phosphine
MC: Microcrystalline cellulose
ECD: Electronic circular dichroism
MS: Mass spectrometry
MALDI: Matrix-assisted laser desorption/ionization
FT-ICR: Fourier-transform ion cyclotron resonance
