# Supplementary material for: The H2O2-dependent activity of a fungal lytic polysaccharide monooxygenase investigated with a turbidimetric assay
Source: Biotechnol Biofuels. 2020 Mar 5;13:37. doi: 10.1186/s13068-020-01673-4 (PMC7057652; doi:10.1186/s13068-020-01673-4)
Supplement: Supplementary file 1 — Additional file 1: Figure S1. Incubation of 3 µM LPMO and 0.8 mg mL−1 PASC with CDH at concentrations of 0.5 µM (red) 1 µM (blue) or 3 µM (green) in absence of cellobiose. Black line: 3 µM CDH and 10 mM cellobiose in absence of LPMO. All reactions were carried out under constant stirring at 30 °C in 50 mM sodium phosphate buffer, pH 6.0. Figure S2. Titration of oxidized LPMO (3 µM) and 0.8 mg mL−1 PASC with 20 µM (green), 40 µM (red) or 80 µM (blue) H2O2 (solid lines). Dashed, coloured lines show the titration of 2 mM ascorbate and 0.8 mg mL−1 PASC with 20 µM (green), 40 µM (red) or 80 µM (blue) H2O2. The vertical dashed lines indicate the addition of H2O2. The arrow indicates the addition of LPMO (solid lines) or 2 mM ascorbate (dashed lines). All reactions were carried out under constant stirring at 30 °C in 50 mM sodium phosphate buffer, pH 6.0. Figure S3. Titration of LPMO (3 µM) and 0.8 mg mL−1 PASC with 40 µM H2O2. The vertical dashed lines indicate the addition of H2O2. The arrow indicates the addition of fresh PASC which was either added alone (green line) or simultaneously with 1 mM ascorbate (AscA, black line). The blue line indicates the addition of ascorbate (1 mM). All reactions were carried out under constant stirring at 30 °C in 50 mM sodium phosphate buffer, pH 6.0. [file 13068_2020_1673_MOESM1_ESM.docx]

**ADDITIONAL INFORMATION**

The H_2_O_2_-dependent activity of a fungal lytic polysaccharide monooxygenase investigated with a turbidimetric assay

**Frantisek Filandr^1,2,3^, Petr Man^1^, Petr Halada^1^, Hucheng Chang^3^, Roland Ludwig^3^ and Daniel Kracher^3,4^**

^1^ BioCeV – Institute of Microbiology, The Czech Academy of Sciences, Prumyslova 595, 252 50 Vestec, Czech Republic

^2^Faculty of Science, Charles University, Hlavova 2030/8, Praha 2, 128 43, Czech Republic

^3^ Biocatalysis and Biosensing Research Group, Department of Food Science and Technology, BOKU - University of Natural Resources and Life Sciences, Muthgasse 18, 1190 Vienna, Austria

^4^The University of Manchester, Manchester Institute of Biotechnology, M1 7DN Manchester, U.K.

Running title: *H_2_O_2_-dependent activity of LPMO*

To whom the correspondence should be addressed: Daniel Kracher (E-mail: danielkracher@boku.ac.at; Telephone: +43 1 47654-75251)

**Keywords:** lytic polysaccharide monooxygenase, cellobiose dehydrogenase, glucose oxidase, hydrogen peroxide, cellulose, *Neurospora crassa*

**Figure S1**. Incubation of 3 µM LPMO and 0.8 mg mL^-1^ PASC with CDH at concentrations of 0.5 µM (red), 1 µM (blue) or 3 µM (green) in absence of cellobiose. Black line: 3 µM CDH and 10 mM cellobiose in absence of LPMO. All reactions were carried out under constant stirring at 30 °C in 50 mM sodium phosphate buffer, pH 6.0.


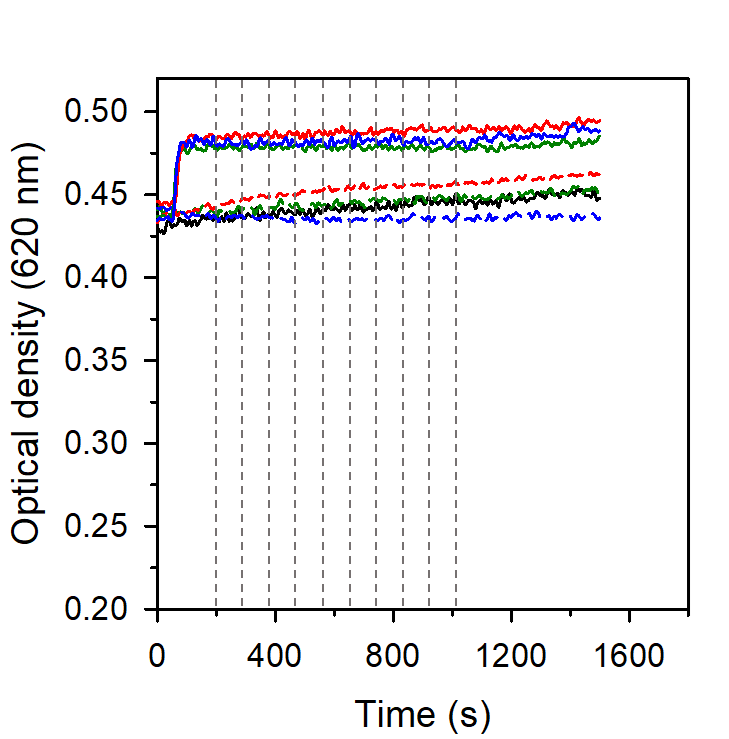


**Figure S2**. Titration of oxidized LPMO (3 µM) and 0.8 mg mL^-1^ PASC with 20 µM (green), 40 µM (red) or 80 µM (blue) H_2_O_2_ (solid lines). Dashed, coloured lines show the titration of 2 mM ascorbate and 0.8 mg mL^-1^ PASC with 20 µM (green), 40 µM (red) or 80 µM (blue) H_2_O_2_. The vertical dashed lines indicate the addition of H_2_O_2_. The arrow indicates the addition of LPMO (solid lines) or 2 mM ascorbate (dashed lines). All reactions were carried out under constant stirring at 30 °C in 50 mM sodium phosphate buffer, pH 6.0.


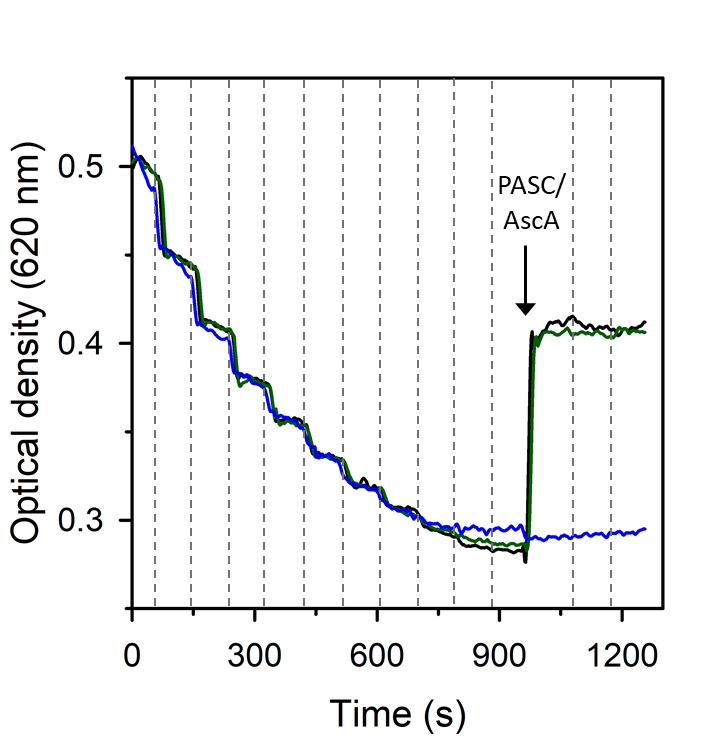


**Figure S3**. Titration of LPMO (3 µM) and 0.8 mg mL^-1^ PASC with 40 µM H_2_O_2_. The vertical dashed lines indicate the addition of H_2_O_2_. The arrow indicates the addition of fresh PASC which was either added alone (green line) or simultaneously with 1 mM ascorbate (AscA, black line). The blue line indicates the addition of ascorbate (1 mM). All reactions were carried out under constant stirring at 30 °C in 50 mM sodium phosphate buffer, pH 6.0.
